# Latitude shapes diel patterns in insect biodiversity

**DOI:** 10.1098/rsbl.2024.0622

**Published:** 2025-04-30

**Authors:** Mark K. L. Wong

**Affiliations:** ^1^ School of Biological Sciences, The University of Western Australia, Perth, Australia

**Keywords:** biodiversity, community, diurnal, cathemeral, nocturnal, global

## Abstract

The writings of naturalists from two centuries past are brimming with accounts of the stark differences in the kinds and numbers of organisms encountered during the day and night as well as between the tropical and temperate zones. However, only recently have ecologists begun to systematically explore the geographic variation in the diel activity patterns of species on Earth. Examining data from 60 insect communities distributed globally, I find that the proportion of nocturnal species in a community declines from a peak of 36% at the equator to 8% at 60° latitude, while the proportion of diurnal species shows no significant trend. By contrast, the proportion of cathemeral (day- and night-active) species in a community increases poleward from 18% to 68% along the same gradient. These latitudinal trends in the partitioning of diel activity time among co-occurring insect species in communities broadly reflect previously documented biogeographic patterns in the global distributions of vertebrate species occupying different temporal niches. Since diel activity patterns shape insect community dynamics, uncovering their mechanistic basis and the roles of factors such as temperature, light and biotic interactions is vital for curbing insect declines in the Anthropocene.

## 1. Introduction

*The moths in certain families, such as the Zygaenidae, various Sphingidae, Uraniidae, some Arctiidae and Saturniidae, fly about during the day or early evening, and many of these are extremely beautiful, being far more brightly coloured than the strictly nocturnal kinds*.’

     – Charles Darwin, 1871, *The Descent of Man, and Selection in Relation to Sex* [1]

‘*The nearer we approach the tropics, the greater the increase in the variety of structure, grace of form, and mixture of colours, as also in perpetual youth and vigour of organic life*.

      – Alexander von Humboldt, 1807, *Views of Nature* (1850 translation) [2]

The contrasting identities and traits of animals active during the day and night were certainly not lost on the early naturalists [1]. Ecological studies have since revealed that the partitioning of diel activity time among such diurnal and nocturnal species in a community can serve as an important coexistence mechanism [3–5]. Today, there is growing interest in the diel variation of biodiversity-mediated ecosystem functions such as pollination, herbivory and nutrient cycling [6]. Furthermore, there is widespread concern that communities occupying specific temporal niches are disproportionately susceptible to the varying levels of anthropogenic light, noise and chemicals in environments over the 24 h diel cycle [7–9].

Despite growing appreciation for the diel dynamics of biodiversity, as well as mounting evidence that cathemerality (activity during both day and night) is a distinct and prevalent diel strategy across the animal kingdom [6,10], there remains a dearth of basic information on the respective proportions of diurnal (exclusively day-active), nocturnal (exclusively night-active) and cathemeral (day- and night-active) species within communities [11]. This problem—symptomatic of the diurnal habits of ecologists [12]—is arguably most severe for insects [13], a functionally diverse and increasingly imperilled group [14].

Beyond noting changes in animal activity over the diel cycle, pioneering naturalists were captivated by the staggering increments in the numbers of species they encountered while journeying from temperate zones towards the tropics [2]. Following in their footsteps, modern ecologists have sought to systematically document and explain latitudinal patterns in species richness [15,16] and more recently in functional diversity [17], genetic diversity [18] and biotic interactions [19].

Given that key factors influencing diel activity such as temperature and light also vary with latitude [11], the proportions of species occupying different temporal niches in a locality may likewise depend on its distance from the equator. Indeed, recent efforts to map the global distributions of diurnal, nocturnal and cathemeral mammals (>4400 species [6,11]) and lizards (>3500 species [20]) across major biogeographic regions revealed broad latitudinal trends. They found that the richness of both nocturnal and diurnal species peaked in the tropics and declined poleward, while that of cathemeral species was highest at mid-to-high latitudes (approx. 40°−70°) [6,11,20]. However, since these patterns were based on the compilation of individual vertebrate species’ distributions across major biogeographic regions, they do not directly reflect how species coexisting within local ecological communities partition diel time. Furthermore, it is unclear whether similar trends apply to invertebrate biodiversity. Hence, there is a need to examine if and how the latitudinal gradient shapes the proportions of species occupying different temporal niches (such as diurnal, nocturnal and cathemeral species) in invertebrate communities globally.

To survey diel activity patterns in insect communities, I searched the literature for studies which had systematically sampled insect communities in comparable day and night periods using methods that exclusively targeted active insects. Multiple studies identified during the search did not report sample-based estimates of species richness and had to be excluded from a subsequent meta-analysis, which mainly examined diel patterns in insect abundance [13]. Many studies, however, did report the total number of species collected across all samples and, importantly, distinguished between species found exclusively in day samples, exclusively in night samples, and those found in both. Based on these empirical observations, I apply a broad classification of diel activity patterns to determine the proportions of species in communities using three main temporal niches: diurnal, nocturnal and cathemeral. Nonetheless, I recognize that other activity patterns exist (e.g. crepuscular species), and that species’ activity can vary along a continuum and be more flexible than the categories suggest. In this paper, I summarize patterns of diel partitioning observed in insect communities, and present preliminary evidence to suggest that these patterns are significantly structured by the global latitudinal gradient.

## 2. Material and methods

### (a) Data compilation

Relevant studies which systematically sampled insect communities across the diel cycle were identified in a literature search on 28 April 2022. The full details of the procedure were described in Wong & Didham [13]; a summary is provided here. The search terms used were ‘(insect) AND (community OR communities) AND (activity OR diel OR nocturnal OR diurnal OR night OR day)’. Only data from studies using sampling methods that collected active individual insects, such as movement-based interception traps (e.g. pitfall traps, sticky traps, malaise traps, drift nets) and some attraction-based traps (e.g. dung-baited pitfall traps) was included. Data from studies using methods which potentially collected inactive individuals (e.g. sweep-netting, beating) as well as methods for which collection efficiency or attractiveness was influenced by environmental changes across the diel cycle, such as light traps and coloured pan-traps, were excluded. Data from a study was only included if the investigators systematically sampled an insect community using the same collection method during the day and the night, and separated the samples collected from each diel period. This allowed for a clear distinction of the species collected during different diel periods, and meant that species’ diel activity patterns were directly observed from the time of collection (in contrast to being indirectly inferred from other information, e.g. specific phenotypic traits). Across all studies that were identified as relevant, the day was considered the period after sunrise and before sunset, while the night was the period after sunset and before sunrise. For each relevant study, I recorded the total number of species collected across all samples, the numbers of species occurring exclusively in day samples (diurnal species), night samples (nocturnal species) and in both day and night samples (cathemeral species). Besides data on community composition, the season(s) during which sampling occurred as well as the elevation and geographic coordinates of the sampled locality were recorded. Using the geographic coordinates, data for seven environmental variables were extracted from the WorldClim database [21] within a 1000 m radius of the locality: the mean annual temperature (BIO1), temperature seasonality (BIO4), minimum temperature (BIO6), maximum temperature (BIO5), precipitation seasonality (BIO15), annual precipitation (BIO12) and solar radiation.

### (b) Data analysis

The influence of latitude and other environmental factors on the proportions of (i) diurnal, (ii) nocturnal and (iii) cathemeral species in the sample of insect communities was investigated using generalized linear mixed models (GLMMs) with a binomial family and logit link function. For each of the three response variables, a null model was built which included only the random effects of a publication identifier and the taxonomic constitution of the community (i.e. an insect order). Next, three different candidate models with the same random effects structure as the null model were constructed to examine the effects of latitude (measured in decimal degrees). The first model included as a fixed effect the absolute value of latitude, the second the raw value and the last the quadratic term. A comparison of the AIC values of each candidate model with that of the null and the other candidate models was then performed to determine if and specifically how variation in the proportion of species using a given diel period exhibited a distinct latitudinal trend. For any relationships that were detected, the influence of other environmental factors was explored by building a candidate model with the same random effects structure with the environmental variable included as a fixed effect, and once again comparing its AIC value to the others. Marginal *R*^2^ [22] was used to determine the amount of variance explained by the fixed terms. Models were built using the *lme4* package and results were visualied using the *ggplot2* package in R software version 4.3.0 [23].

## 3. Results

**Figure 1.**
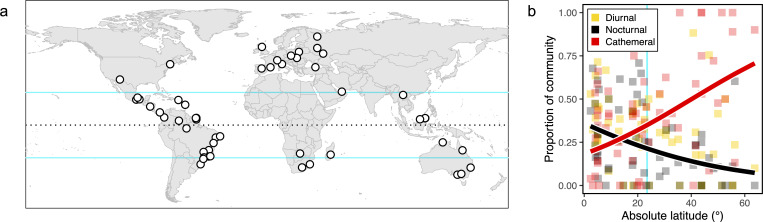
The latitudinal gradient influences the proportions of species occupying different temporal niches in insect communities. (a) The global distribution of the 60 insect communities used in the study. Dotted line indicates the equator, blue lines demarcate the tropical zone. (b) The proportions of species occupying three temporal niches in insect communities distributed along a gradient of absolute latitude. Plot shows individual data points for the proportions of diurnal, nocturnal and cathemeral species in the communities. With increasing distance from the equator, the proportion of nocturnal species in a community (black regression line) decreases significantly and the proportion of cathemeral species (red regression line) increases significantly, while that of diurnal species does not vary significantly. Vertical blue line indicates tropical zone.

Data on the diel activity patterns of species in 60 insect communities from 50 studies were compiled, and these included multiple communities of the most diverse insect orders, Coleoptera, Diptera and Hymenoptera (electronic supplementary material). The studied localities were distributed globally and had a relatively even span across the tropical and temperate latitudinal zones (−35.7−63.7) (figure 1a). Sampling effort varied considerably across the studies (e.g. >9000 dung-baits in [24] versus 24 flight-interception traps in [25]), however, the different collection methods meant that sample sizes were not comparable indicators of sampling effort or sample coverage among the studies.

There was high variation among the communities in total species richness (*M* = 36.2, s.d. = 45.5), as well as in the relative proportions of species occupying the three different temporal niches. On average, diurnal species comprised 31% (s.d. = 20%) of total species. The proportions of nocturnal and cathemeral species showed greater variability, averaging 24% (s.d. = 21%) and 43% (s.d. = 31%) of total species, respectively. The proportion of cathemeral species in a community strongly negatively correlated with the proportions of diurnal (*r* = −0.71) and nocturnal (*r* = −0.73) species, which were weaky correlated with one another (*r* = 0.07).

The latitudinal gradient significantly influenced the proportions of species occupying different temporal niches in insect communities (figure 1b). A significant negative relationship was observed between the proportion of nocturnal species and absolute latitude (model ‘N.lat.1’, marginal *R^2^* = 0.19, ΔAIC = −6.0). The predicted proportion of nocturnal species was 36.0% (CI: 23.1−51.2%) at the equator, decreasing to 18.2% (CI: 12.5−25.8%) as latitude increased to 30° and 8.1% (CI: 3.6−17.1%) at 60° latitude (figure 1b). By contrast, a significant positive relationship was observed between the proportion of cathemeral species and absolute latitude (model ‘C.lat.1’, marginal *R^2^* = 0.13, ΔAIC = −3.4). The predicted proportion of cathemeral species was 18.2% (CI: 6.7−41%) at the equator, increasing to 40.6% (CI: 22.0−62.4%) at 30° latitude and 67.7% (CI: 35.6−88.9%) at 60° latitude (figure 1b). The proportion of diurnal species in a community did not vary significantly along the latitudinal gradient.

**Figure 2.**
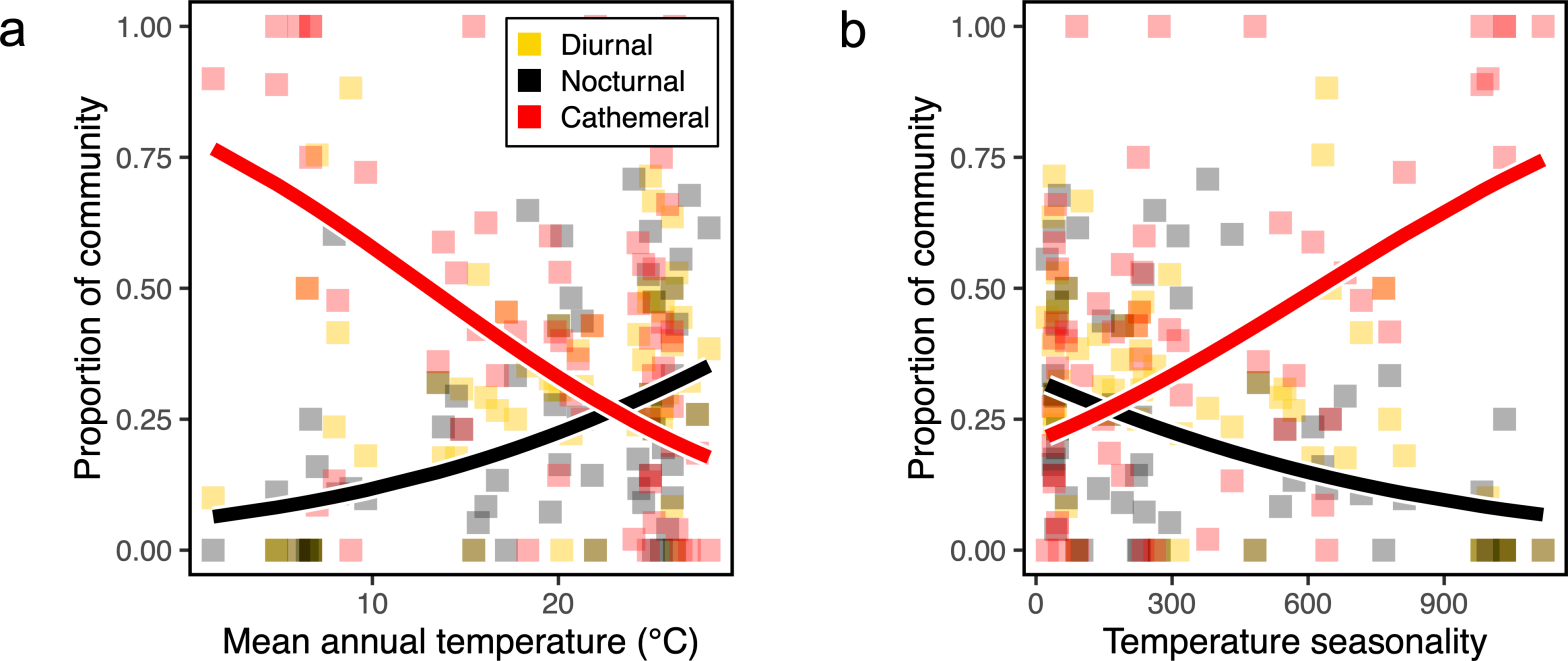
The proportions of species occupying three temporal niches in insect communities distributed along gradients in mean annual temperature (a) and temperature seasonality (b). Plots show individual data points for the proportions of diurnal, nocturnal and cathemeral species in insect communities. Regression lines illustrate the significant relationships in the proportions of nocturnal species (black) and cathemeral species (red) across the two climatic gradients.

Among separate models which included different variables of temperature, precipitation and solar radiation as individual predictors (electronic supplementary material), two variables best accounted for variation in both the proportions of nocturnal and cathemeral species in communities: the mean annual temperature and the extent of temperature seasonality in the surrounding environment (figure 2). Both variables showed strong correlations with latitude: mean annual temperature (*r* = −0.95) and temperature seasonality (*r* = 0.91). Moreover, the contrasting directional trends for the proportions of nocturnal versus cathemeral species in communities along these climatic gradients broadly reflected those observed with latitude. Specifically, the proportion of nocturnal species increased with increasing mean annual temperature (model ‘N.env.1’, marginal *R^2^* = 0.23, ΔAIC = −8.0) and decreased with increasing temperature seasonality (model ‘N.env.4’, marginal *R^2^* = 0.21, ΔAIC = −5.2), while the proportion of cathemeral species decreased with increasing mean annual temperature (model ‘C.env.1’, marginal *R^2^* = 0.17, ΔAIC = −5.4) and increased with increasing temperature seasonality (model ‘C.env.1’, marginal *R^2^* = 0.15, ΔAIC = −4.6) (figure 2).

## 4. Discussion

Despite their importance to ecosystem dynamics, how patterns of diel partitioning in insect communities vary across biogeographic gradients is still not well understood. The present findings highlight the variability in these patterns globally, and reveal a significant relationship with latitude, where poleward declines in the proportions of nocturnal species in communities appear to be compensated by increments in the proportions of cathemeral species. I must impress that the generality of these relationships are tempered by the small sample, which provides limited representation of global insect diversity, and the heterogeneous data sourced from unrelated studies, which used a variety of sampling protocols not originally intended for comparative analysis. Nonetheless, it is interesting that the relationships observed at the community level for insects (figure 1b) broadly mirror the diverging poleward trends in the richness of nocturnal and cathemeral mammal and lizard species across biogeographic regions globally [6,11,20]. These consistent patterns across both vertebrate and invertebrate groups, spanning different ecological scales, suggest that geography—particularly the latitudinal gradient—plays a key role in shaping the activity patterns of organisms across the diel cycle.

The precise mechanisms underpinning the observed latitudinal variation in the diel partitioning of insect community composition are unclear. Based on this preliminary investigation, the decrease in environmental temperatures with latitude is likely to play a role, given the diverging trends in the proportions of nocturnal and cathemeral species found in communities distributed along a gradient in mean annual temperature (figure 2a), which reflect the diverging trends also observed with latitude (figure 1). As ectotherms, insects predominantly use behavioural strategies rather than physiological ones to thermoregulate, modifying their activity in response to environmental temperature [26]. At low latitudes, consistently high daytime temperatures often approximate the upper thermal limits of insect species [27]. Therefore, the peak proportions of species displaying nocturnal activity in tropical communities may represent an effective behavioural strategy for thermoregulation in these warmer environments. Conversely, nocturnality may be energetically costly in temperate regions where night temperatures approximate species’ lower thermal limits [28]. To cope with these constraints at higher latitudes, nocturnal lineages may have shifted to cathemeral or diurnal activity, which is afforded by the relatively moderate daytime temperatures regionally.

In addition to shifting mean temperatures, changes in levels of temperature seasonality with latitude may also influence community diel activity patterns. This is supported by the similar trends observed along the latitudinal gradient and the temperature seasonality gradient, which are tightly correlated; that is, decreasing proportions of nocturnal species and increasing proportions of cathemeral species with increasing temperature seasonality and latitude (figure 2b). It is possible that at high latitudes, more seasonal and variable temperatures may limit the adaptation to strictly diurnal or nocturnal strategies. By contrast, cathemeral species with comparatively flexible activity patterns may be better poised to exploit temporally variable climates for resource acquisition and reproduction.

In conjunction with temperature, geographic variation in light regimes may also shape diel activity patterns. While solar radiation levels did not significantly explain geographic variation in the diel activity patterns of insect communities in this analysis (electronic supplementary material), it is possible that solar radiation is too coarse a measure to be ecologically relevant for many insect species. Moreover, Bennie *et al*. [11] found that the richness of mammal species occupying different temporal niches within a biogeographic region strongly correlated with the specific duration of the diel cycle during which illumination by natural light coincided with environmental temperatures that fell within the thermal limits of mammalian activity (0−35°C). Future studies could therefore model global variation in such periods of ‘biologically useful light’ for insects, based on taxon-specific thermal limits, to assess whether this better explains latitudinal patterns in the diel partitioning of insect communities. While it remains unclear whether the same factors—temperature and light—that explain biogeographic patterns in mammalian diel strategies [11] also drive latitudinal patterns in insect community diel partitioning (figure 1), the role of biotic interactions at the community level also warrants consideration.

Beyond the abiotic factors explored in this study, patterns of diel partitioning in communities may be shaped by species interactions such as competition and predation which vary with latitude. It has been suggested that interspecific competition for resources is more intense in tropical communities [15]. If so, such intense competition may promote greater partitioning of diel activity time among tropical species, resulting in high proportions of diurnal and nocturnal specialists, and low proportions of more generalist cathemeral species that are less well adapted to daytime or nighttime conditions. In comparison, temperate communities, which are typically characterized by lower diversity and less intense competition, may provide more opportunities for cathemeral species to exploit resources throughout the diel cycle. Notably, interspecific competition has regularly been cited as key driver of diel partitioning in tropical communities of ants, bees and dung beetles (e.g. [29–31]). Besides competition, latitudinal variation in predation risk, which has been shown to peak in the tropics, especially among insects [19], may also have a role to play. In particular, the high diversity of nocturnal insects in tropical regions may represent a strategy to avoid diurnal predators. For instance, the richness of Lepidoptera with strictly nocturnal larvae in the Amazon has been linked to the demonstrably lower attack rates by predators during the night [32]. Notably, tropical regions often support dense vegetation and complex microhabitats, which can be exploited by nocturnal insects as refuges from diurnal predators during the day [33]. By contrast, temperate habitats may have more open and less structurally complex landscapes that afford fewer refuges for inactive nocturnal species during the day.

There is fertile ground for exploring the mechanistic basis and macroecology of the diel partitioning of community composition. Importantly, the latitudinal trends presently observed (figure 1) are in need of hard evidence from systematic studies of diel variations in species richness, abundance and ecological interactions replicated across multiple regions globally. Complementary insights may also be acquired through similar work along elevational gradients, which mirror many aspects of environmental variation along the latitudinal gradient [34]. If the patterns hold true, an exciting next step will be to compare the relative contributions of different hypothetical mechanisms. For instance, future studies experimentally manipulating temperature and light levels in controlled mesocosms or in the field (e.g. by installing shade canopies) in both tropical and temperate ecosystems may test the influence of these factors on diel patterns. Similar investigations into the roles of competition and predation can be achieved by manipulating levels of resource availability, or the densities of competitors and predators across tropical and temperate ecosystems, and examining how such treatments alter the activity patterns of focal species in different regions.

The diel activity patterns of organisms and their interactions are key to biodiversity and ecosystem functioning, yet they may be increasingly threatened by anthropogenic changes to temperature and light. With much still unknown about these patterns, today’s ecologists must continue the work of the early naturalists—deciphering the intricate rhythms of animal behaviour.

## Data Availability

Data and code for reproducing the analysis is attached as electronic supplementary material. Supplementary material is available online [35].
